# The power of instruction on retropulsion: A pilot randomized controlled trial of therapeutic exercise focused on ankle joint movement in Parkinson’s disease

**DOI:** 10.1016/j.prdoa.2022.100151

**Published:** 2022-07-01

**Authors:** Ryoma Taniuchi, Toshihide Harada, Hiroaki Nagatani, Takako Makino, Chigusa Watanabe, Shusaku Kanai

**Affiliations:** a Department of Rehabilitation, National Hospital Organization Hiroshima-Nishi Medical Center, Hiroshima, Japan; b Graduate School of Comprehensive Scientific Research, Prefectural University of Hiroshima, Hiroshima, Japan; c Department of Physical Therapy, Faculty of Health and Welfare, Prefectural University of Hiroshima, Hiroshima, Japan; d Department of Neurology, National Hospital Organization Hiroshima-Nishi Medical Center, Hiroshima, Japan

**Keywords:** Exercise therapy, Parkinson’s disease, Postural balance, Randomized controlled trial, Rehabilitation

## Abstract

**Introduction:**

Although retropulsion is a serious complication of Parkinson’s disease (PD), it is unknown whether ankle joint movement patterns can be targeted to treat retropulsion. The primary aim of this study was to investigate the effectiveness of therapeutic exercise focused on instructions regarding ankle joint movement on retropulsion in PD.

**Methods:**

Twenty patients with moderate PD were randomly allocated to the experimental intervention (INSTR) or control groups. The INSTR group received a 2-week therapeutic exercise program (40 min/day, five times/week) that involved repeated backward pulls on the shoulders with instructions to land on the toes as a response, and the control group received the same intervention without the instructions. The primary outcome was the difference in changes from baseline in the Movement Disorder Society-sponsored revision of the Unified Parkinson’s Disease Rating Scale (MDS-UPDRS part III) score between the study groups at weeks 1 and 2.

**Results:**

The improvement in the MDS-UPDRS part III scores was significantly greater for the INSTR group in the week 1 (p = 0.033, _p_η^2^ = 0.241) and week 2 (p = 0.004, _p_η^2^ = 0.401) assessments. However, the provision of instructions to land on the toes as a backward response induced improvement in the only scores related to the backward response, and no significant between-group differences were observed in the other outcomes.

**Conclusion:**

The 2-week therapeutic exercise program with instructions to treat retropulsion improved the backward response.

**Trial registration:**

UMIN-CTR, UMIN000042722.

## Highlights

• Retropulsion in PD may involve the lack of push-off for a backward step.
• Exercise with ankle-movement instruction can improve backward response.
• Toe-landing instruction may facilitate treatment of retropulsion in PD.

## 1 Introduction

Although postural instability is a common and severe complication of Parkinson’s disease (PD), the effects of dopaminergic medications on postural instability are generally negligible [1], [2]. Patients with PD mostly fall forward (46% of all falls), while 20% of their falls are backward [3]. However, patients with PD are more vulnerable to backward falls [4], which lead to fall-related fractures of the femur and an increased risk of hospital admission [5], [6]. The likelihood of a forward fall may be increased by the typical stooped posture because the associated forward shift of the center of gravity (COG) provides relative protection against a backward fall [7], [8]. Therefore, effective and feasible therapeutic exercises to treat retropulsion are needed to avoid the debilitating consequences of falls in PD.

Recently published meta-analyses have shown that incorporating components addressing balance dysfunction into therapeutic exercise regimens effectively improves postural instability in PD [9], [10]. However, the current evidence does not clarify the primary component of retropulsion requiring treatment. An interesting therapeutic exercise for retropulsion in PD is based on the use of repetitive compensatory steps, which improve the length of compensatory steps and step initiation [11]. This repetitive training appears to treat balance dysfunction by applying motor learning principles. A biomechanical study of retropulsion in patients with PD in comparison with healthy controls identified two different ankle joint movement patterns in response to a backward balance disturbance [12]: (1) at liftoff, patients with PD showed ankle dorsiflexion, whereas healthy controls showed ankle plantarflexion, (2) patients with PD had an abnormal ankle dorsiflexion orientation (i.e., heel landing) that was a disadvantage to push-off force generation for a backward step during balance recovery. However, it is unknown whether ankle joint movement patterns can be targeted to treat retropulsion in patients with PD.

Incorporating repetitive step training and the biomechanical findings that underlie retropulsion into a therapeutic exercise should offer a new avenue for treatment. The primary aim of this pilot study was to investigate the effectiveness and feasibility of therapeutic exercise that focuses on ankle joint movement instructions for retropulsion in patients with moderate PD. We hypothesized that a 2-week therapeutic exercise program with instructions to correct postural stability and motor learning would positively influence backward response.

## 2 Methods

### 2.1 Trial design

This study was a single-blinded randomized controlled trial (RCT). Eligible participants were randomly allocated (in a 1:1 ratio) into one of two groups through the permuted block method (block sizes of 2 or 4) using computer-generated random number codes. After randomization, the participants were only informed about the allocated exercise regimen for their group and were not provided details about the differences between the exercise regimens for both groups. The experimental intervention (INSTR) group received a 2-week therapeutic exercise program involving repeated backward pulls on the shoulders with instructions to land on the toes as a response. The control group received the same intervention but without the instructions. The control group also received the therapeutic exercise program with instructions after the study period to ensure fair treatment.

### 2.2 Participants

Patients with idiopathic PD and postural instability clinically diagnosed by a neurologist were recruited from a national hospital. The inclusion criteria were as follows: (1) Modified Hoehn and Yahr scale score [13] of 2.5–4, (2) inpatient treatment, and (3) ability to walk independently with or without a walking aid. The exclusion criteria were as follows: (1) deep brain stimulation surgery, (2) dementia (Mini-Mental State Examination score < 24), and (3) uncontrolled chronic conditions that would interfere with the safety and conduct of the exercise. The study was approved by the National Hospital Organization Hiroshima-Nishi Medical Center’s Committee of Ethics in Research (No. H30-015), and all participants provided written informed consent before enrollment. The trial registration number was UMIN000042722.

### 2.3 Interventions

A certified physical therapist in neuromuscular disorders was responsible for administering the therapeutic exercise to all patients during the study period. The daily sessions lasted 40 min and consisted of 25 min of warm-up followed by 15 min of therapeutic exercise. Weekends were excluded, so the therapeutic exercise was performed five times a week for 2 weeks in an “ON medication” state. Warm-up included current physical therapy [14], [15] such as aerobic exercise (5 min), stretching (10 min), and high-amplitude movements (5 min) as well as active workouts for muscular power and posture (5 min).

The therapeutic exercise consisted of repetitive backward pulls on the patient’s shoulders by the physical therapist. The physical therapist stood behind the patient and explained that they could take a step backward to avoid falling. The pull force was sufficient to displace the COG, at least to a degree requiring the patient to take a step backward. The instructions on landing on the toes as a backward response were given orally before the pulls and numerous times during the therapeutic exercise. Only the non-dominant PD side was used to perform the compensatory backward step because PD tended to be less consistent in the choice of stepping limb [12]. We hypothesized that the non-dominant PD side would be easier to perform the stepping than the dominant PD side. Preliminary stepping limb determination will provide a way to avoid hesitation. The dominance of parkinsonism was determined by the methodology described by Uitti et al. [16], which uses the difference in the absolute value of the right-sided and left-sided scores on the Movement Disorder Society-sponsored revision of the Unified Parkinson’s Disease Rating Scale (MDS-UPDRS) part III [17], items 3–8 and 15–17. Patients with a difference of one or more absolute values were determined to be asymmetric. If the differences were symmetrical and the dominant PD side was unclear, the handedness side was considered the dominant PD side [18]. Positive feedback was provided for patients who showed satisfactory compensatory steps on toe landing, and the pull force gradually increased. In contrast, for those who showed inadequate compensatory steps on heel landing, toe landing was enforced by oral instruction again, the force of the pulls was reduced, if necessary, and somatosensory cueing was given to the toe by tapping [14], [19].

In the second half of the intervention, the instructions on toe landing were reduced, and sudden unexpected pulls were performed randomly. Patients in the control group received the same therapeutic exercise program consisting of repetitive backward pulls on the shoulders, but they were not instructed on how to land on their toes as a backward response. Positive feedback was given if a stepping response to recapture the falling body could be executed regardless of the result of the toe landing. In both groups, compensatory backward steps were provided with sufficient challenge and performed as often as possible within each 15-minute exercise session. For safety, the physical therapist remained ready to catch the patients.

### 2.4 Outcome measures

Demographic and clinical data, including medication history, were collected at baseline (T0). The primary efficacy outcome was the intergroup difference in the MDS-UPDRS part III score change from T0 to week 1 (T1) and week 2 (T2). MDS-UPDRS part III scores were subdivided into four subscores according to cardinal motor symptoms: tremor (sum of the scores for items 15–18), rigidity (item 3), bradykinesia (sum of the scores for items 2, 4–9, and 14), and axial (sum of the scores for items 1 and 10–13) [20]. Patients were recorded on video for blinded assessment of the backward response while conducting the pull test to evaluate postural instability on the MDS-UPDRS part III, item 12. Videos of the pull test were then rated by an experienced neurologist blinded to the group assignment and time point of assessment. The pull test remains the most widely known technique and can easily test for postural stability in PD in a clinical setting, mainly because its performance and interpretation are relatively simple and do not require specific instruments. Although the utility and technical performance of the pull test has been controversial [21], any abnormal score is correlated with a risk of falls in moderate-to-severe PD [22]. Surprisingly, even if PD patients fall on the first pull, they do not show a learning effect when pulled multiple times in the same direction [23]. To minimize differences in the performance of the pull test, the baseline assessment and assessments at each subsequent time point were executed by the same trained examiner. Moreover, the secondary outcomes included timed up and go (TUG), fast walking speed (assessed by a 10-m walk), and performance in activities of daily living (ADL) as measured by the Barthel Index. All assessments were performed in the “ON medication” state.

### 2.5 Statistical analysis

Intergroup differences in baseline demographic and clinical characteristics were analyzed using the Mann-Whitney *U* test or chi-square test because of the non-normal distribution of data. Analysis of covariance (ANCOVA) with the baseline values as a covariate was performed to compare the significance of intergroup differences in the changes in MDS-UPDRS part III scores from T0 to T1 and T2. The Friedman test was applied to analyze within-group changes in the outcomes over time (from T0 to T2) for the intervention effect. If the overall effect from the Friedman test was significant, post hoc evaluations were performed using Wilcoxon signed-rank tests between T0 and T1 and between T0 and T2.

The same analysis was performed for the secondary outcomes. Statistical analyses were conducted using IBM SPSS Statistics version 26 (IBM Corp., Armonk, NY, USA), with the level of significance set at 5%. A priori power analysis indicated that at least 52 participants were required to detect an effect size (f) of 0.40 (power = 80%, α-level = 0.05). The analysis was conducted using G*Power version 3.1.9.6; Heinrich-Heine-Universität Düsseldorf, Düsseldorf, Germany (http://www.gpower.hhu.de). However, after analyzing 20 patients, the effect size (_p_η^2^) for the MDS-UPDRS part III was 0.401 (f = 0.818), and the power was 93%, indicating that the statistical goal had already been met. Therefore, 20 patients were included in the present study.

## 3 Results

**Fig. 1:**
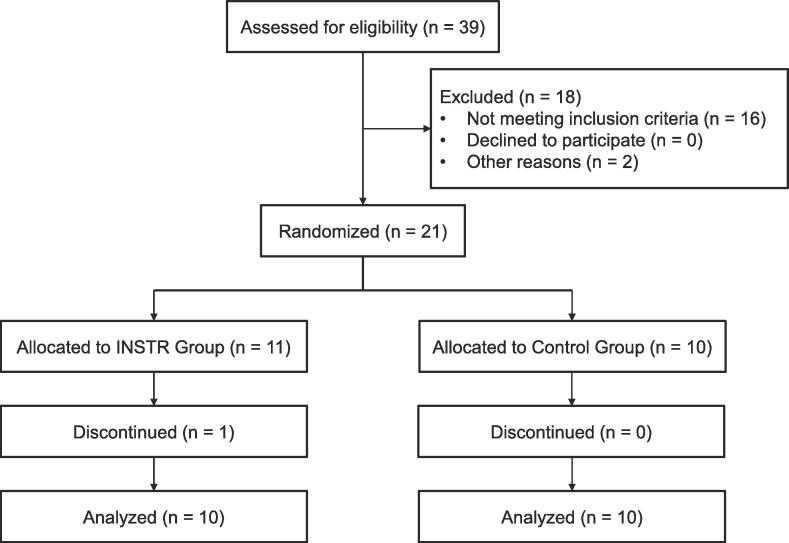
Flow diagram of the study participants.

Of the 21 patients, 10 each in the INSTR and control groups completed the 10 exercise sessions. One patient in the INSTR group discontinued the interventions due to clinical depression (Fig. 1). Throughout the study period, no adverse effects or falls were observed. The intergroup differences in demographic and clinical characteristics and the outcome measures at T0 were not significant (Table 1, Table 2). Regarding the pharmacological effect, adjustments of dopaminergic medication between T0 and T2 were performed in one patient in the INSTR group. However, the changes in the L-dopa equivalence dose (LED) resulting from the adjustments were negligible (+50.0 mg/day) and did not differ significantly (Wilcoxon signed-rank test, p = 0.317).

**Table 1:** Baseline demographic and clinical characteristics of the study groups.

	INSTR (n = 10)	Control (n = 10)	p-value
Gender (Male/Female)^b^	5/5	5/5	> 0.99
Age (years)^a^	76.2 ± 6.4	73.5 ± 8.2	0.529
Modified H-Y^b^			0.809
Stage 2.5, n (%)	2 (20.0)	1 (10.0)	
Stage 3, n (%)	5 (50.0)	6 (60.0)	
Stage 4, n (%)	3 (30.0)	3 (30.0)	
Disease duration (years)^a^	7.3 ± 2.8	7.4 ± 3.6	0.739
MMSE^a^	26.6 ± 2.4	27.2 ± 2.3	0.631
People who fell in the past year, n (%)^b^	5 (50.0)	6 (60.0)	0.653
Dominant hand (Right/Left)^b^	10/0	7/3	0.060
Occupational therapy intervention, n (%)^b^	9 (90.0)	9 (90.0)	> 0.99
Medications			
Daily levodopa equivalent dose (mg)^a^	380.0 ± 141.8	430.0 ± 133.7	0.315
Participants receiving dopamine agonists, n (%)^b^	8 (80.0)	7 (70.0)	0.606
Participants receiving MAO type B inhibitors, n (%)^b^	2 (20.0)	2 (20.0)	> 0.99
Participants receiving COMT inhibitors, n (%)^b^	3 (30.0)	1 (10.0)	0.264
Participants receiving amantadine, n (%)^b^	1 (10.0)	0 (00.0)	0.305
Participants receiving droxidopa, n (%)^b^	1 (10.0)	1 (10.0)	> 0.99
Participants receiving zonisamide, n (%)^b^	4 (40.0)	4 (40.0)	> 0.99

Data are expressed as mean ± SD unless stated otherwise.

Abbreviations: H-Y, Hoehn and Yahr; MMSE, Mini-Mental State Examination; MAO, monoamine oxidase; COMT, catechol-*O*-methyltransferase.

^a^Mann-Whitney *U* test.

^b^Chi-square test.

**Table 2:** Results of the primary and secondary outcomes.

	T0 (baseline)	T1 (week 1)	T2 (week 2)	ANCOVA between group	
				ΔT0-T1	ΔT0-T2
	Mean ± SD	Mean ± SD	Mean ± SD	F/p-value^‡^ (_p_η^2^)	F/p-value^‡^ (_p_η^2^)
**Primary outcomes**					
MDS-UPDRS part III scores					
Total score^†^					
INSTR	38.4 ± 11.4	28.8 ± 8.9^**^	23.1 ± 10.3^**^	5.4/0.033* (0.241)	11.4/0.004^**^ (0.401)
Control	39.0 ± 13.6	32.4 ± 12.2^**^	28.1 ± 11.2^**^		
Tremor subscore^†^					
INSTR	2.9 ± 2.6	1.7 ± 2.2*	1.5 ± 1.5*	0.1/0.808 (0.004)	0.5/0.477 (0.030)
Control	2.0 ± 2.0	1.0 ± 1.5	0.8 ± 1.2		
Rigidity subscore^†^					
INSTR	8.9 ± 3.7	7.7 ± 3.4*	6.9 ± 3.1*	0.2/0.689 (0.010)	0.2/0.634 (0.014)
Control	8.2 ± 3.5	7.3 ± 3.1*	6.7 ± 3.0*		
Bradykinesia subscore^†^					
INSTR	18.6 ± 5.0	14.5 ± 4.4^**^	11.5 ± 5.8^**^	2.1/0.170 (0.108)	0.8/0.384 (0.045)
Control	19.8 ± 8.6	16.8 ± 7.3*	13.6 ± 7.1^**^		
Axial subscore^†^					
INSTR	8.0 ± 2.8	4.9 ± 2.6^**^	3.2 ± 2.2^**^	5.0/0.040* (0.226)	16.9/< 0.001^***^ (0.498)
Control	9.0 ± 2.5	7.3 ± 3.3*	7.0 ± 3.1^**^		
Pull test^†^					
INSTR	2.7 ± 0.7	1.0 ± 1.2*	0.1 ± 0.3^**^	1.5/0.231 (0.083)	12.3/0.003^**^ (0.419)
Control	2.4 ± 0.8	1.5 ± 1.4*	1.4 ± 1.3*		
**Secondary outcomes**					
TUG (s)^†^					
INSTR	17.86 ± 9.23	13.02 ± 6.10^**^	12.12 ± 5.48^**^	1.5/0.239 (0.081)	0.1/0.744 (0.006)
Control	18.84 ± 10.89	15.66 ± 7.77	13.10 ± 5.68*		
Fast walking speed (m/s)^†^					
INSTR	0.89 ± 0.28	1.10 ± 0.38*	1.22 ± 0.34^**^	1.0/0.341 (0.054)	1.9/0.188 (0.100)
Control	0.90 ± 0.30	1.04 ± 0.34	1.10 ± 0.43		
Barthel Index^†^					
INSTR	82.5 ± 10.6	–	92.5 ± 10.6*	–	1.4/0.248 (0.078)
Control	78.0 ± 14.2	–	85.5 ± 12.6*		

Abbreviations: ANCOVA, analysis of covariance; MDS-UPDRS, Movement Disorder Society-sponsored revision of the Unified Parkinson’s Disease Rating Scale; TUG, timed up and go.

^†^Significance levels of *p < 0.05 and ^**^p < 0.01 in intragroup comparisons (T0 vs. T1 or T0 vs. T2) using the Wilcoxon signed-rank test.

^‡^Significance levels of *p < 0.05, ^**^p < 0.01, ^***^p < 0.001 in ANCOVA.

### 3.1 Primary outcomes

**Fig. 2:**
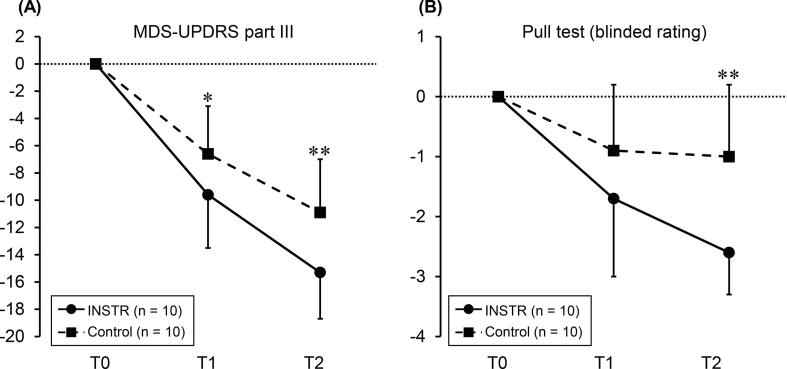
(A) MDS-UPDRS part III score: mean change from T0. (B) Pull test score (blinded rating): mean change from T0. Compared with that in the control group (interrupted line), the change between T0 and T2 was superior in the INSTR group (solid line); *p < 0.05, ^**^p < 0.01 in ANCOVA. MDS-UPDRS, Movement Disorder Society-sponsored revision of the Unified Parkinson’s Disease Rating Scale; ANCOVA, analysis of covariance.

The primary outcomes are presented in Table 2. ANCOVA showed significant intergroup differences in the changes in MDS-UPDRS part III scores from T0 to T1 (F = 5.4, p = 0.033) and T2 (F = 11.4, p = 0.004; Fig. 2-A). The mean change from T0 to T2 was −15.3 ± 3.4 in the INSTR and −10.9 ± 3.9 in the control groups. Similarly, the pull test rating improvement for blinded assessment was significantly greater in the INSTR group than in the control group at T2 (ANCOVA, F = 12.3, p = 0.003; Fig. 2-B). Concerning the subscores, only the improvement in the axial subscore was significantly greater in the INSTR group than in the control group from T0 to T1 (ANCOVA, F = 5.0, p = 0.040) and T2 (ANCOVA, F = 16.9, p < 0.001). Overall, a group-dependent improvement in the MDS-UPDRS part III scores from T0 to T2 was observed in both groups (Friedman test, INSTR: p < 0.001; control: p < 0.001).

### 3.2 Secondary outcomes

ANCOVA did not reveal significant intergroup differences in the changes from T0 to T2 for TUG test results, fast walking speed, and Barthel Index (Table 2). In multiple comparisons, group-dependent improvements in TUG test results (Wilcoxon signed-rank test, INSTR: p = 0.005; control: p = 0.037) and the Barthel Index (Wilcoxon signed-rank test, INSTR: p = 0.011; control: p = 0.017) from T0 to T2 were noted in both groups. Fast walking speed was significantly increased only in the INSTR group (Wilcoxon signed-rank test, p = 0.005).

## 4 Discussion

This study investigated the effectiveness and feasibility of therapeutic exercise focusing on instruction of ankle joint movement for treating retropulsion in patients with moderate PD. A total of 20 patients completed the planned exercise sessions, and no adverse effects or falls occurred. These results suggest that therapeutic exercise is safe and feasible for patients with moderate PD in the short term. Furthermore, interference due to the adjustments of the LED was not observed. A few previous studies have suggested that dopaminergic medication does not improve the stepping response in PD [1], [2], and the present results strengthen our assertion that therapeutic exercise has an independent effect.

The main findings of this study were that ANCOVA showed significant group differences in the MDS-UPDRS part III scores. The instruction on landing on the toes as a backward response induced improvements only in the scores related to the backward response, such as the axial subscore and the pull test score. This significant change in the backward response with a large effect size (f = 0.818) was considered clinically relevant. Even though ANCOVA did not reveal significant intergroup differences in the changes from T0 to T2, positive intervention effects were observed in only the tremor subscore and fast walking speed for the INSTR group. The reduction of ankle push-off force is related to the decreased walking speed of patients with PD [24]. The INSTR group could apply repetitive push-off as toe landing and improve the walking speed than that observed in the control group. However, regression to the mean effects cannot be ruled out due to baseline differences between the groups.

To our best knowledge, only a few intervention studies have focused on a specific factor for the treatment of postural instability in PD [11], and this is the first RCT that focused on abnormal ankle joint movement as the primary treatment point for retropulsion. The ankle dorsiflexion orientation (i.e., heel landing) observed in patients with PD at retropulsion may be a disadvantage because no push-off force for a backward step can be generated during balance recovery [12]. The reason for inability of patients with PD to generate a push-off force during backward response is presumably the difficulty in switching ankle joint movements due to excessive co-contraction in the ankle muscles [25]. Compared with healthy controls, patients with PD showed enhanced activity in the premotor cortex and cerebellum when performing a movement than when in an automatic state [26], and hyperactivation in these regions is recognized as compensation for dysfunction of the basal ganglia. In addition, the cortico-motoneuronal connection is stronger in the tibialis anterior muscle than in the soleus muscle [27], which may induce excessive background activity in the tibialis anterior muscle [4]. These previous findings could partially explain why heel landing at retropulsion occurs in patients with PD and why therapeutic exercise for patients with PD should focus on the abnormal ankle joint movement to compensate for the specific defective pathokinesiological mechanisms underlying postural instability.

In this study, two factors may have contributed to improving the backward response in patients with PD. First, cognitive engagement may be enhanced by instructions on toe landing. In PD, verbal instructions or cues that encourage attention to therapeutic exercise practice may strengthen cognitive engagement, facilitating the modification of the learned abnormal movement [28]. Patients with PD tend to use abnormal anticipatory postural adjustments (APAs) before compensatory step initiation due to dysfunction of the supplementary motor area [12]. In contrast, goal-directed control guided by sensory cueing may bypass the dysfunction of the APAs associated with regions such as the supplementary motor area or the basal ganglia [19], [28], [29], [30]. Thus, for patients with PD, the instruction on toe landing to guide appropriate ankle joint movements could offer a kinesiological advantage over the backward response initiated by an internally and abnormally generated ankle joint movement, such as heel landing. Second, motor learning theories such as repetitive behavioral experience is a potent modulator of brain plasticity. However, Nieuwboer et al. suggested that the effectiveness of motor learning in PD is limited as the disease progresses, and these limitations should be accommodated using explicit motor learning methods and augmented sensory input [30]. Crucially, patients with PD have great difficulty developing habitual control despite repeated practice [23], which strongly suggests that an increase in motor activity without goal-directed control is insufficient to enhance neuroplasticity [29]. Therefore, the results of this study support the notion that a therapeutic exercise that incorporates specific goal-directed motor learning improves backward response in PD and that this might be facilitated through cognitive engagement such as instruction [28].

This pilot study had several limitations. First, this clinical trial was conducted at a single center with a relatively homogenous patient group with respect to age, disease severity, and geographic location, which makes the effects of therapeutic exercise insufficient to be generalized to all patients with PD. The participants were limited to patients who could be hospitalized for 2 weeks or longer to exclude the interferences of ADL as much as possible, which subsequently affected the representative nature of the sample used in this study. Moreover, many nonpharmacological treatments are subject to potential confounders, such as adherence to the treatment as intended [31]. For example, considering the nature of this clinical trial, active patients for whom therapeutic exercise is potentially effective may have been attracted to participate in this study. Second, the findings were also affected by the limitations of the pull test. It is controversial whether using the test in an experimental situation is sufficient to change the actual circumstances in which falls occur in daily life. Thus, the participants may have only learned a limited aspect of the backward response to avoid falling. Further multicenter studies with a larger number of participants are needed to establish whether therapeutic exercise focusing on instruction of ankle joint movement is associated with long-term fall-preventing effects and the changes in specific pathophysiological mechanisms underlying retropulsion in the PD population.

## 5 Conclusion

Our results suggest that providing instructions on toe landing may be an important component for treating retropulsion in PD. Because instruction, irrespective of the type of exercise, is a non-invasive, virtually risk-free treatment option, a combination of therapeutic exercise and instruction should be regarded as an essential treatment for postural instability in PD.

### CRediT authorship contribution statement

**Ryoma Taniuchi:** Conceptualization, Data curation, Formal analysis, Investigation, Methodology, Project administration, Supervision, Writing – original draft, Writing – review & editing. **Toshihide Harada:** Conceptualization, Formal analysis, Methodology, Project administration, Supervision, Writing – original draft, Writing – review & editing. **Hiroaki Nagatani:** Data curation, Investigation, Methodology, Project administration, Writing – review & editing. **Takako Makino:** Data curation, Investigation, Methodology, Project administration, Writing – review & editing. **Chigusa Watanabe:** Data curation, Methodology, Project administration, Writing – review & editing. **Shusaku Kanai:** Methodology, Project administration, Supervision, Writing – review & editing.

## Declaration of Competing Interest

The authors declare that they have no known competing financial interests or personal relationships that could have appeared to influence the work reported in this paper.
